# Acknowledgement to Reviewers of *Cells* in 2013

**DOI:** 10.3390/cells3010150

**Published:** 2014-02-25

**Authors:** Cells Editorial Office

**Affiliations:** MDPI AG, Klybeckstrasse 64, CH-4057 Basel, Switzerland; E-Mail: cells@mdpi.com

The editors of *Cells* would like to express their sincere gratitude to the following reviewers for assessing manuscripts in 2013:

Ahmed, Zubair
Altan-Bonnet, Nihal
Anderson, Kurt I.
Aravalli, Raj
Auffray, Charles
Barnes, Chris P.
Baumgarth, Nicole
Bergmann, Andreas
Bilodeau, Steve
Bizzarri, Mariano
Burnette, Dylan T
Cain, Kelvin
Cedersund, Gunnar
Cereghetti, Grazia
Chao, Sheau-chiou
de Bernard, Marina
Decrock, Elke
Desai, Kaushik M.
Di Bernardo, Diego
Driscoll, Paul C.
Dziegielewski, Jaroslaw
Emmert-Streib, Frank
Flores, Ana I.
Fölsch, Heike
Frade, José M.
Grégoire, Marie-Chantal
Gu, Yangnan
Guo, B.
Gurevich, Vsevolod V.
Gutierrez-Arenas, Omar
Herman, Ira M.
Hoflack, Bernard
Holtz, William
Jacot, Jeff
Jauberteau, Marie-odile
Jeffery, Glen
Joshi, Ravindra P.
Kandouz, Mustapha
Kleinerman, Eugenie S.
Kobayashi, Yoshiro
Kobeissy, Firas H.
Kürten, Stefanie
Lavrik, Inna N.
Law, Ping-yee
Lee, Won-Ha
Li, Tieshi
Lin, Jin-Ming
Lin, Nianwei
Liu, Kebin
Lu, N. Z.
Ma, Liang
Mann, Matthias
Matsubara, Etsuro
Maus, Carsten
Mauvais-Jarvis, Franck
Meda, Paolo
Meng, Qingying
Meyer, Jerrold
Miklavcic, Damijan
Minden, Audrey
Mironov, Alexandre
Miyoshi, Eiji
Neutzner, Albert
Noble, Denis
Nuccitelli, Richard
Panfoli, Isabella
Plotkin, Lilian I.
Pollard, David
Prasanth, Supriya G.
Prince, Lynne
Psarra, Anna-maria G.
Rodriguez-Fernandez, Maria
Romanel, Alessandro
Ropella, Glen
Rosslenbroich, Bernd
Roti Roti, Joseph
Silke, John
Silvestre, Samuel
Simons, Kai
Singh, Ishwar S.
Smith, Leslie S.
Sundararaman, Srividya
Suski, Jan M.
Szallasi, Arpad
Tabata, Keiichi
Tabata, Yasuhiko
Tam, Emily W.Y.
Tang, Chuan Y.
Teasdale, Rohan D.
Tian, Ruijun
Tran-Son-Tay, Roger
Vortkamp, Andrea
White, Anne
Woodman, Philip
Yang, Wan-xi
Yano, Ken-ichi
Zaidel-Bar, Ronen
Zal, Tomasz
Zampese, Enrico
Zhang, Chaoyang
Zhang, Tongli
Zhou, Huarong
Zhou, Lei
Zhou, Qinghua

